# Comparing artistic and geometrical perspective depictions of space in the visual field

**DOI:** 10.1068/i0668

**Published:** 2014-10-16

**Authors:** Joseph Baldwin, Alistair Burleigh, Robert Pepperell

**Affiliations:** Cardiff School of Art & Design, Cardiff Metropolitan University, Cardiff, CF5 2YB, UK; e-mail: rpepperell@cardiffmet.ac.uk

**Keywords:** visual field, visual space, geometrical perspective, art, space perception, wide-angle vision

## Abstract

Which is the most accurate way to depict space in our visual field? Linear perspective, a form of geometrical perspective, has traditionally been regarded as the correct method of depicting visual space. But artists have often found it is limited in the angle of view it can depict; wide-angle scenes require uncomfortably close picture viewing distances or impractical degrees of enlargement to be seen properly. Other forms of geometrical perspective, such as fisheye projections, can represent wider views but typically produce pictures in which objects appear distorted. In this study we created an artistic rendering of a hemispherical visual space that encompassed the full visual field. We compared it to a number of geometrical perspective projections of the same space by asking participants to rate which best matched their visual experience. We found the artistic rendering performed significantly better than the geometrically generated projections.

## 1 Introduction

Which is the most accurate way to depict space in our visual field? The normal human binocular visual field extends some 180° horizontally (Strasburger, Rentschler, & Jüttner, 2011). The vertical extent can vary depending on individual facial features and expression, but is around 130° when the head is still and the eye axes are parallel (Lachenmayer & Vivell, 1992). The “visual field” is distinct from the “field of view,” which is the region of space visible as the eyes move around in their sockets while the head is still (Pirenne, 1970, p. 35). The visual field is composed from the views of two laterally displaced eyes, which fuse to form an apparently unified image (Ogle, 1964).

Most depictions of the visible world in paintings, drawings, photographs, and computer graphics represent only a limited section of this visual field (Hagen, Jones, & Reed, 1978). This may be in part because people are not necessarily aware of its full scope; Koenderink, van Doorn, and Todd (2009) found great variation in the extent of the apparent visual field among observers presented with a 180° visual space, with most perceiving it as being closer to 90°. Under certain circumstances, however, it may be desirable or necessary to depict much larger portions of the binocular visual field or field of view, up to or beyond 180°. Artists, for example, have tried to represent the expanse of a landscape or cityscape, or the enveloping space of an architectural interior (Davies, 1992; Flocon & Barre, 1987; Gayford, 2007; Hansen, 1973; Herdman, 1853; Parsey, 1836). Designers of head mounted displays and virtual reality systems may also wish to represent the entire visual field to create a more natural or immersive experience (Keller & Colucci, 1998).

Depicting the appearance of the visual world in the full scope of the visual field is challenging. Artists and architects have recognised the problems associated with representing three-dimensional space on a two-dimensional plane for centuries (Alberti, 1991; Kemp, 1990). The traditional method of achieving this, linear perspective, is impractical when very wide angles of view are concerned because the correct viewing position, the centre of perspective (Kingslake, 1992) or centre of projection (Kubovy, 1986), is usually too close to the picture surface to be seen properly. For example, representing a horizontal visual field of 164° using a camera with a standard 36 mm full frame sensor and rectilinear lens would require a lens with a focal length of 2.5 mm, which is impractically short in many circumstances. The angle of visual field can be calculated with the formula:

V=2arctan(S/2f),

where *V* is the angle, *S* is sensor size, and *f* is focal length of the lens. Enlarging the image to, say, 1,000 mm in width puts the centre of perspective, and therefore the correct viewing distance, at just 70 mm from the picture surface. The correct viewing distance can be calculated with the formula:

$$D=f(\frac{W}{S}),$$

where *D* is viewing distance, *f* is focal length of the lens, *W* is picture width, and *S* is sensor size. Viewed from a greater distance perspective “distortion” will become apparent (Kingslake, 1992). By enlarging the picture the viewing distance can be increased, but there are obvious practical limitations. The problem becomes more acute the more the angle approaches 180°; to create an image with an angle of view of 180° would require an infinitely small focal length.

Alternatives to rectilinear camera lenses are available for capturing wide angles of view. Fisheye lenses, for example, can span 180° or greater (Nikon produced a 6 mm lens that spanned 220°) but they introduce severe “barrel distortion,” which looks unnatural (Ying, Hu, & Zha, 2006). Stereographic projection has been proposed as a superior alternative to fisheye perspective since it preserves the shape of depicted objects more faithfully (Fleck, 1994). Panoramic methods can also be used to capture wide visual fields, and are normally constructed by stitching together multiple shots. But they typically result in very tall or wide image formats, and this can make them undesirable in many situations (Shum & Szeliski, 2000). There are a number of other projections that can be used to represent wide angles of view, many of which were developed for cartography and astronomy where spherical spaces need to be mapped onto two-dimensional planes. Among them are Mercator, Sinusoidal, Equisolid, and Pannini. Each projection will distort different aspects of the space being depicted, and so each has its advantages and disadvantages depending on the application and on which spatial properties the user wishes to preserve (Maling, 1992; Sharpless, Postle, & German, 2010).

Our research addresses the problem of how to represent the full extent of the visual field in pictures that are viewable from normal distances and without excessive distortion. We have developed a method derived from careful mapping of the contents of the visual field through painting and drawing (Pepperell, 2012). It is based on direct observation rather than geometrical or optical principles, and depends on the artist exercising judgment in order to measure and record the perceived size, shape, and position for objects in the scene. In a previous study we showed pictures created with this method represent visual space in a way that deviates consistently from the rules of linear perspective. Artists such as John Constable, Vincent van Gogh, and Paul Cézanne used a broadly similar approach when painting landscapes, as did a group of people with art training when drawing a still life (Pepperell & Haertel, 2014). But to date no studies have been carried out to determine whether artistic depictions are superior to their geometrical perspective counterparts in terms of being able to accurately convey the visual space they depict. Nor has there been any evaluation of the effectiveness of artistic methods as a means of representing the full scope of the visual field.

In the present study we created an artistic depiction of a space that encompassed the entire visual field and made a number of geometrical perspective depictions of the same space from the same station point. These images were then shown to participants, who had to select which most accurately represented their visual impression of the space using a scale of 1 (very low) to 5 (very high). We took into account a number of factors that might have influenced the results, including participants' age, gender, and whether or not they had corrected vision. On the basis of our previous work we hypothesised the artistic depiction would more accurately represent the appearance of the space than the geometrical perspective projections.

## 2 Methods

### 2.1 Creating an artistic depiction of the visual field

**Figure 1.**
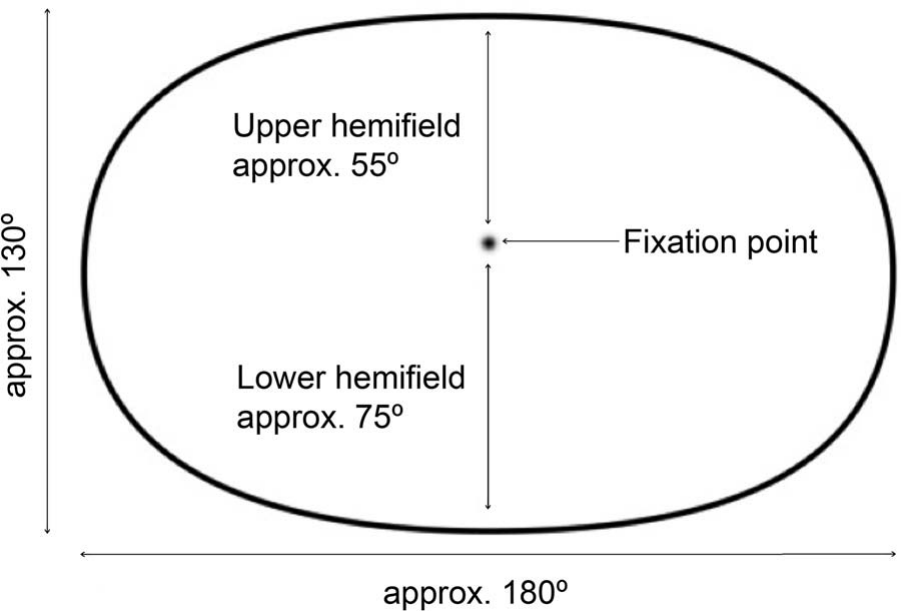
Elliptical picture space approximating the shape and extent of the binocular visual field, represented as a cyclopean image that fuses the area visible to both eyes when looking ahead at the fixation point. The fixation point is located closer to the top of the field, reflecting the anatomical structure of the human eyes and face.

The general method of creating artistic representations of the visual field begins by defining an elliptical picture space that approximates the dimensions and shape of the human visual field (Figure 1). An elliptical boundary for the picture space is used because, as Gibson (1950) noted, it is closer to the shape of the visual field than the conventionally used rectangle. A fixation point is chosen in the scene, normally directly ahead of the viewer, and the equivalent point plotted in the picture space, this being located slightly above the horizontal centre, which reflects the fact that the human eye sees more in the lower part of the visual field than the upper (Lachenmayer & Vivell, 1992). The contents of the visual field are then mapped onto the picture space such that the boundaries of both coincide. Judgements are made about the portion of the visual field occupied by each perceived object, its location and shape, and these are recorded in the drawing. Having carried out this procedure many times in relation to different scenes we have found it consistently results an image in which the area of the scene being viewed in central or foveal vision is enlarged compared to how it would appear in an equivalent rectilinear or fisheye perspective projection (Pepperell & Haertel, 2014; Pepperell, in press. See also Figure 4). The degree of enlargement applied in each case depends on a number of factors, including the size of objects being depicted, their proximity to the viewing station, and the distance between the artist and the depiction as it is being made.

**Figure 2.**
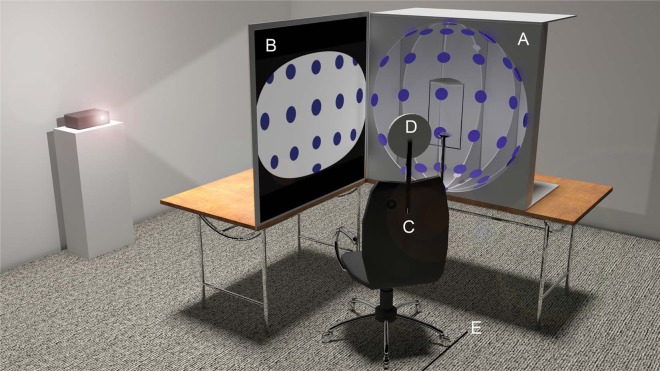
Illustration of the apparatus used to create the artistic rendering of the visual field and the subsequent experiments. On the right table is the hemispherical dome (A) containing a series of blue discs, showing the chinrest and headrest, and on the left is the rear projection screen (B) on which depictions of the visual space inside the hemisphere were projected and drawn. The swivel chair (C) allowed the artist or participant to move between the view of the dome and the screen while the head restraint (D) ensured they were in the correct viewing position. The chair was positioned by markers on the floor (E).

**Figure 3.**
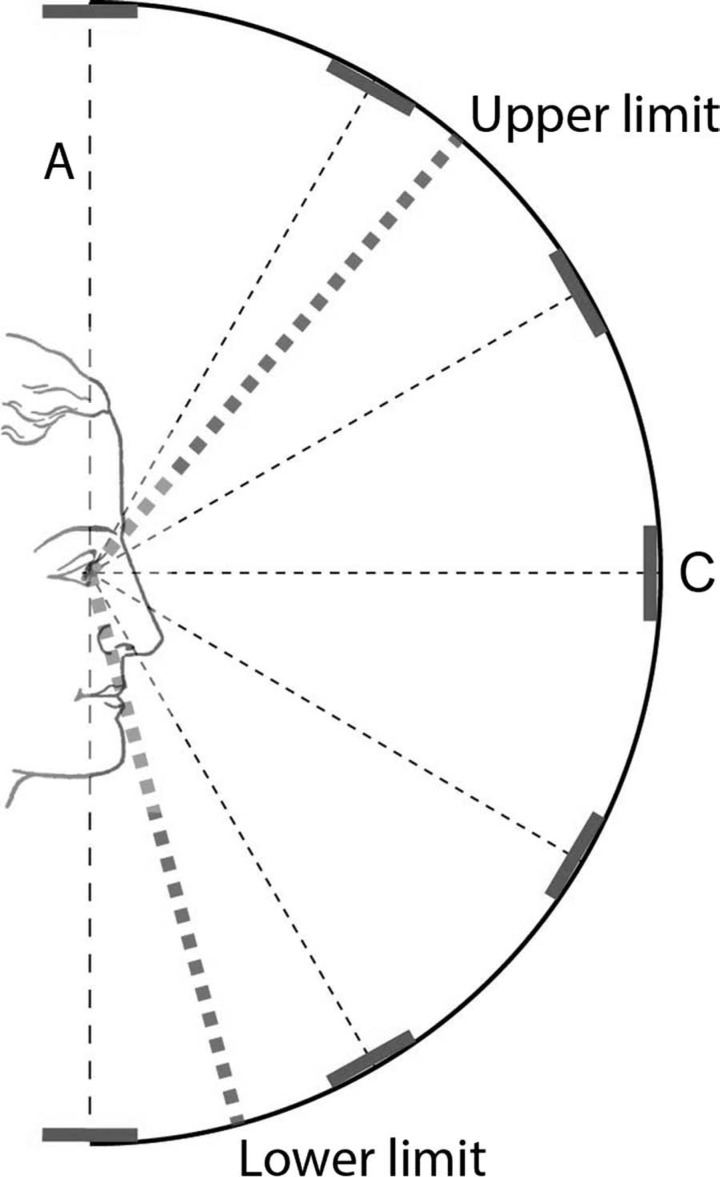
An illustration of the position of the viewer in the dome apparatus, shown in side-view cross-section. The eyes are in line with the central disc, labelled C. The dotted line labelled A shows the position of an opaque screen that obscured the view of the discs prior to the artists being properly positioned in the apparatus. The upper and lower limits of the visual field are indicated. The eyes of the viewer are located 450 mm from the central disc C.

To create the specific artistic depiction used in this study, and to conduct the subsequent experiments, we constructed a concave hemispherical dome of 900 mm diameter (Figure 2). Inside the dome we placed a number of blue discs, each 75 mm in diameter. The discs were arranged such that each vertical row was separated by 30° of longitude and each horizontal row was separated by 30° of latitude. A chinrest and a forehead restraint were mounted in the dome to ensure the viewers placed in the apparatus had an eye-line view directly opposite the central disc (see Figure 3). An opaque screen with an aperture for the head was stretched across the open side of the dome to block the view of the discs until the viewers were properly positioned in the apparatus. Its purpose was to minimise the influence of seeing the arrangement of discs from any position other than the one specified. The centre point between the viewers' eyes was located equidistantly from all the discs, i.e. 450 mm. In this position the binocular visual field of the viewers was fully encompassed by the apparatus.

Placed next to the dome at 90° was a rear projection screen of the same horizontal width as in the dome (900 mm) and a projector mounted behind the screen set up to project images of equivalent width. The projector was carefully positioned and keystoned to ensure the projected image was completely central and straight. The viewers were seated on a revolving chair that allowed them to swap between the viewing position in the dome and viewing the screen. When viewing the screen, a headrest on the chair and markers on the floor ensured the viewers' eyes were located at a distance of 450 mm from the screen. Indirect lighting sources were used to ensure there were no shadows in the dome, and the light levels between the dome and the screen were equalised using a Sekonic light meter.

**Figure 4.**
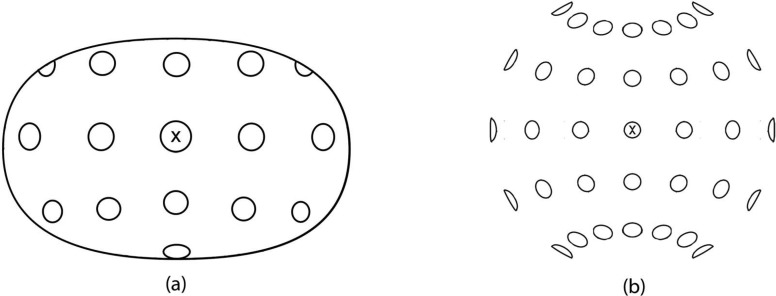
An artistic depiction (a) and a fisheye perspective projection (b) of evenly spaced and equally sized discs in the hemispherical dome. Both pictures were generated from the same station point 450 mm from, and in direct line with, the central fixation point, marked X.

To make the drawing the artist studied the space from the secured position in the dome, fixating on the central disc while forming a mental image of the space as a whole. Judgements were made about the portion of visual field occupied by the each visible disc, their location and shape, and these were then recorded by drawing on an Apple Macintosh computer using Adobe Illustrator and a computer mouse, the results of which were projected on the screen in a picture space framed by an elliptical boundary. By swapping back and forth between the dome and the screen the artist compared and adjusted the depiction until satisfied that the drawing was an accurate representation of the visual field being depicted. Figure 4a shows the layout of the artistic depiction.

In order to create a geometrical perspective projection of the dome we modelled the scene in the three-dimensional animation package Blender and took a “virtual photograph.” This was necessary because the physical bulk of a real camera prevented us from locating its sensor at the same station point as the artist's eye. Figure 4b shows a depiction generated by a virtual 8 mm fisheye lens (equisolid projection) located at the same station point as the artist, i.e. 450 mm from the central disc. In this position the camera captures a 180° angle of view both horizontally and vertically. Fisheye projection was chosen as it captures the full scope of the visual field, unlike a rectilinear perspective projection, which cannot practically accommodate such a wide angle of view.

### 2.2 Stimuli

**Figure 5.**
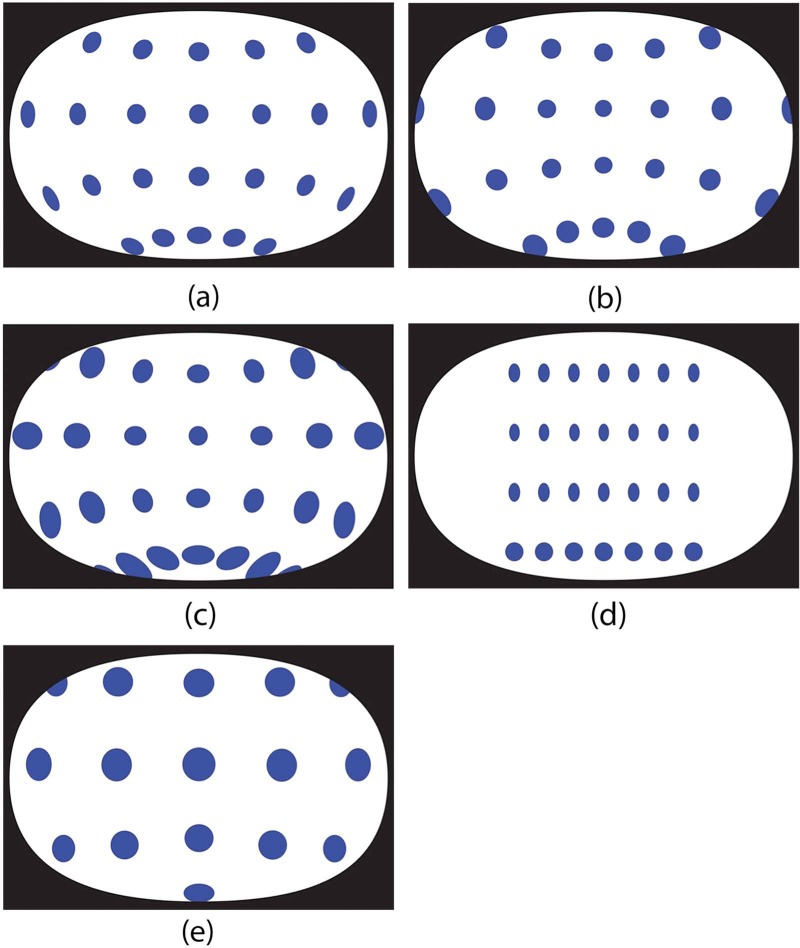
The different projections used as stimuli: (a) Fisheye, (b) Stereographic, (c) Cyclopean, (d) Equirectangular, (e) Artistic.

To compare the accuracy of the different forms of depiction we presented participants with five images on the projection screen, each displayed at 900 mm in width. The images consisted of one artistic and four geometrically generated renderings of the dome space. We chose to show five different renderings in order to make the task of selecting the most accurate depiction more challenging for the participants than it would have been in a two-alternative task, and to enable us to compare the performance of several kinds of geometrical projection. To ensure consistency each depiction was framed in the elliptical boundary approximating the shape and dimensions of the human visual field shown in Figure 1, and cropped according to its upper and lower limits, as shown in Figure 3. The forms of depiction used were as follows (see Figure 5).

Figure 5(a): This stimulus is a monocular fisheye equisolid projection of the scene. It is generated in Blender by a virtual 8 mm fisheye lens located at a point directly in line with the central disc, at the mid-point between the eyes, and at the same distance from the central disc as the participants in the apparatus. Fisheye perspective projection is a common method of capturing very wide visual fields, i.e. > 90° diameter (Fleck, 1994; Kingslake, 1992), in this case representing a view 180° wide.

Figure 5(b): This stimulus is a monocular stereographic projection of the scene, generated using a virtual model of the dome in Blender and the geometric manipulation software PTGui (produced by New House Internet Services B V, Rotterdam). Fleck (1994) argued stereographic projection better preserves the shape and size of objects than fisheye perspective projections and so is a preferable method of representing wide angles of view. Note that the peripheral discs are less squashed in this projection than in the fisheye perspective version.

Figure 5(c): This stimulus is a cyclopean projection of the scene, generated by combining two 8 mm fisheye renderings. It was taken with virtual cameras in Blender located at the same points occupied by a viewer's two eyes in the apparatus, converging on the central disc. These two images were then overlaid to form a single image, which is the composite of both views. The purpose of using this projection was to simulate aspects of the binocular visual field that were necessarily missing from the other monocular geometrical projections. Due to the complexities of binocular vision, however, this can only be an approximate representation (Ogle, 1964).

Figure 5(d): This stimulus is a computer-generated equirectangular 360° projection of the same scene, which is obviously perceptually inaccurate. It was included in the study as a distractor stimulus to detect whether participants were effectively discriminating between the different projections and to make it harder for them to guess the “correct” projection. We anticipated this would be given a low accuracy rating.

Figure 5(e): This stimulus is an artistic rendering of the scene created by observing its appearance when fixating with both eyes on the central disc in the dome. It was drawn on and Apple Macintosh using Adobe Illustrator while the image was projected on the screen viewed at the same distance (450 mm) as the central disc was from the eye. Like stimulus 3, this is a cyclopean rendering that approximates the fused view of the scene produced by binocular vision.

The geometrically generated stimuli used in this study are a subset of the many possible projections of three-dimensional space. But in order to keep the experimental procedure manageable we limited the stimuli to five, two of which are commonly used methods of representing wide angles of view: fisheye perspective and stereographic (Fleck, 1994). We did not use rectilinear perspective projections, because they appear excessively distorted when used to represent very wide angles of view and require impractically close viewing distances. Nor did we use panoramic images, which generate elongated aspect ratios, and would not have fitted within the elliptical picture space used for the rest of the stimuli (Kingslake, 1992).

### 2.3 Experimental procedure

To conduct the experiments we used the same apparatus as shown in Figure 2. First, participants completed a questionnaire to determine gender, age, and whether or not they had corrected vision. They were then given the following instructions about the task:

*Look into the apparatus, keeping your head still, and focus on the centre disk for 30 seconds without looking around. While you are focusing on the central disc, make a mental note of how the whole space appears to you, and try to remember it*.

If glasses were worn the participants were asked to remove them; we did not want the rims to obscure their peripheral visual field when fixating on central disc. They then adopted the same viewing position as the artist, described above, and studied the dome from the specified distance. The opaque screen prevented them forming a visual impression of the scene from any other distance. Having completed this part of the task after 30 seconds, participants were brought out of the apparatus and given further instructions:

*You will now be shown 5 images projected on the screen. View each image and rate how closely it matches the way the space appears to you. Use the scale 1 (very low) to 5 (very high). Before you look at each image you can look into the space for ten seconds to refresh your memory of how it appears*.

Each participant looked into the space for a further 10 seconds, and then returned to the seated position in front of the screen. The experimenter ensured the correct position was adopted. The participants then freely viewed one of the five stimuli and rated how closely it matched their visual impression of the physical space on the five-point scale. The rating was recorded and they moved on to the next image in the sequence. A repeated-measures design was used in which the stimuli were shown in two different orders, one the reverse of the other, to ensure there was no effect of the sequence of stimuli. Once they had completed the cycle, the participants were then offered the opportunity to go back and adjust their ratings. Most took this opportunity and altered one or more of the ratings. They were given as long as they needed to do this, and had the option of looking into the space again if necessary. We wanted to ensure participants were satisfied their ratings had been accurately recorded. Using this general procedure we carried out two experiments. Participants were volunteers and were given no prior indication of the purpose of the experiment; no financial reward was offered, and all gave informed consent.

## Experiment 1

In the first experiment we recorded the responses of 14 participants, 11 female and 3 male. The mean age was 25; four needed vision correction.

### 3.1 Results and discussion

A one-way within-subjects ANOVA was conducted on the ratings of how closely each participant matched their visual impression of the space in the dome to that of the stimuli. We found a significant effect of the factor *ratings*: *F*(4,52) = 17.962, *p* < 0.05, partial *n*^2^ = 0.58. We then conducted a Bonferroni post-hoc test that revealed a preference (*p* < 0.05) for stimulus *e* (artistic) over stimulus *b* (stereographic), stimulus *c* (cyclopean) and stimulus *d* (equirectangular). Stimulus *e* (artistic) was preferred to stimulus *a* (fisheye), but the margin was not significant (*p* = 0.095). As expected, stimulus *d* (equirectangular) was rated poorly. A one-way between-subjects ANOVA was conducted on the influence of participants' gender, vision condition, age bracket, and stimuli viewing order on the ratings of the stimuli. This revealed no significant effects (*p* > 0.05).

During the experiment two participants reported seeing an afterimage following their initial 30 second exposure to the discs in the dome, although they did not report this during the shorter exposure times in the comparison stage, nor did they report using the after image to guide their ratings of the stimuli. However, we wanted to eliminate the possibility that participants were, consciously or unconsciously, using the after image of the discs in the dome to judge the disc size on the screen.

## 4 Experiment 2

To minimise the potential influence of after images we replaced the central blue disc in the dome with a light grey one, and modified the stimuli accordingly. Here the chroma and luminance contrast between the disc and the background were lower. According to values obtained from the histogram tool in Adobe Photoshop, the blue disc in the stimuli had a Weber contrast value against the background of approximately −83% (luminance value of blue disc = 43 and background = 255). The grey disc has a Weber contrast value of approximately −18% (luminance value of grey disc = 209). In pilot tests we found the grey disc induced a weak, blurry afterimage that faded more rapidly and was almost entirely invisible when looking at the projection screen. Using these modified materials we reran Experiment 1 with 14 different participants, 10 female and 4 male. The mean age was 35, and eight needed vision correction.

### 4.1 Results and discussion

**Figure 6.**
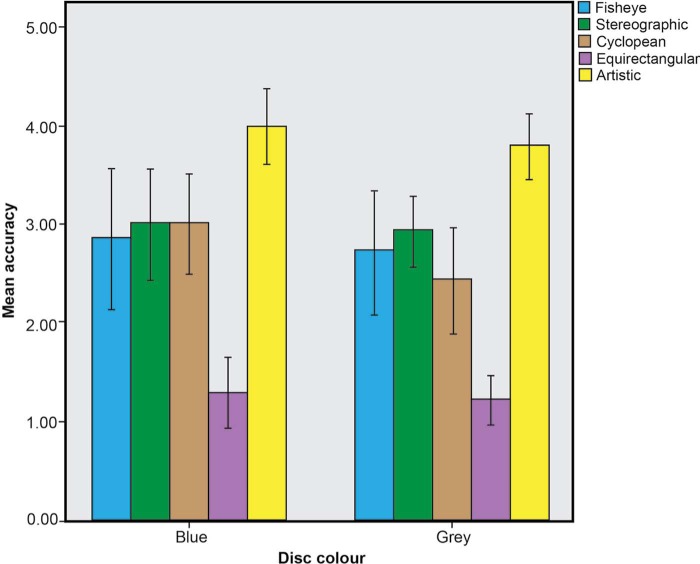
Graph showing the mean accuracy ratings of each stimulus in the blue and grey disc conditions (Error bars: 95% CI).

A one-way within-subjects ANOVA was conducted on the ratings of the stimuli. Again this showed a significant effect of the factor *ratings*: *F*(4,52) = 28.566, *p* < 0.05, partial *n*^2^ = 0.69. A Bonferroni post-hoc test showed a significant preference (*p* < 0.05) for stimulus *e* (artistic) over stimulus *b* (stereographic), stimulus *c* (cyclopean) and stimulus *d* (equirectangular). Stimulus *e* (artistic) was preferred to stimulus *a* (fisheye), but not by a significant margin (*p* = 0.055). Stimulus *d* was again rated poorly. A one-way between-subjects ANOVA was conducted on the influence of participants' gender, age bracket, and stimuli viewing order on the ratings of the stimuli. This revealed no significant effects (*p* > 0.05) apart from an effect of vision condition, with participants who normally wear glasses giving lower ratings to stimuli *a* (fisheye) (*F*(1, 12) = 9.288, *p* < 0.05) and *c* (cyclopean) (*F*(1, 12) = 12.025, *p* < 0.05) than those who did not need vision correction. In order to establish whether there was any differential effect between the blue and grey disc condition we carried out an independent *t*-test. This showed that while the mean value for each stimulus in the blue condition was slightly higher than in the grey condition there was no significant difference between the two (see Figure 6).

**Figure 7.**
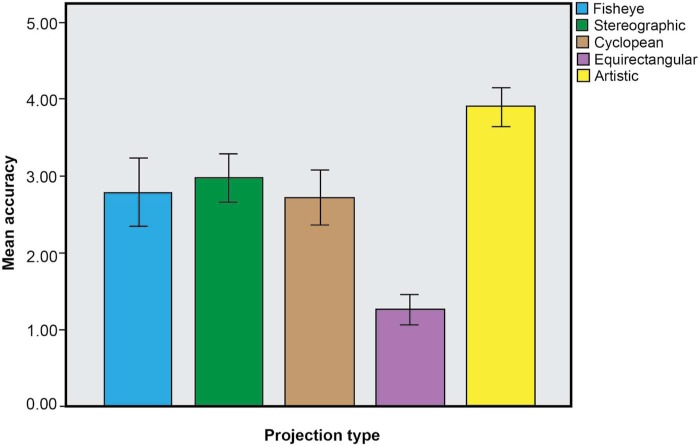
Graph showing the mean accuracy ratings for the combined set of results from Experiments 1 and 2 (Error bars: 95% CI).

Combining the data from both the blue and grey disc conditions (number of participants 28) also showed a significant effect of the factor *ratings*: *F*(4, 108) = 43.913, *p* < 0.05, partial *n*^2^ = 0.62. A Bonferroni post-hoc test showed a significant preference (*p* < 0.05) for stimulus *e* (artistic) compared to all the other stimuli (see Figure 7).

These results show the artistic rendering of the visual space was judged the most accurate depiction by a significant margin. None of the other factors considered, including age, gender, viewing order, disc colour, or condition of vision seems to have influenced the results. The only exception was the lower ratings given to the fisheye (*a*) and the cyclopean (*c*) stimuli by people who needed vision correction in Experiment 2. As we did not record any further details about their vision, such as whether and to what degree they were myopic, we are unable at this stage to attribute this result to any particular condition. It is interesting to note that the performance of the cyclopean depiction (*c*), which as far as we can tell is a novel form of geometrical projection, was on a par with that of the fisheye (*a*) and stereograph (*b*) projections.

## 5 General discussion

We have shown that an artistic depiction represents the appearance of a space encompassing the visual field with greater accuracy than a set of geometrically generated projections. We took into account a number of factors that might have affected the results, including participant age, gender, condition of vision and we controlled for the presence of afterimages and order of stimuli presentation. None of these had a significant influence, with one exception noted above in Experiment 2.

We consider two possible explanations for these results. First, the extent of visual space recorded by the artist, approximately 140° × 100°, was less than the 180° × 130° extent of the visual field visible in the geometrical projections. This indicates that under the viewing conditions in the dome, where fixation was straight ahead on the central disc, the most eccentric discs were not perceived by the artist. If the same was true for the participants this may help to explain why they judged this image to be the most accurate representation of their visual experience in the dome. The fact that a narrower extent of visual field was recorded supports the findings of a study by Koenderink et al. (2009) who presented observers with an array of dots in a hemispherical dome to test the apparent horizontal angle of visual field. Under monocular viewing conditions, where observers were free to move their eye in the experimental apparatus, they found the median reported value to be around 90° rather than the 180° of space that was physically present.

Second, the artistic method records onto a flat surface what Kenneth Ogle called the “subjective world of form, spatial relationships, and color” rather than the objective properties of light stimuli, as recorded by geometrical perspective (Ogle, 1964, p. 10). Linear perspective projections appear “distorted” when wide-angle views are presented on flat surfaces and viewed from outside their centre of projection, and fisheye perspective projections appear distorted from all viewing distances. The artistic method, however, takes the flat surface into account while the scene is being drawn, and so more faithfully preserves the perceived size and layout of objects in the scene.

Many experts have claimed that, because it is based on laws of geometry and the behaviour of light, geometrical perspective is the only accurate way to represent the three-dimensional visual world on a two-dimensional plane (e.g. Gibson, 1971; Gombrich, 1960; Pirenne, 1970; Rehkämper, 2003; Ward, 1976). The job of geometrical perspective, they argue, is not to record how we perceive a given scene but to present the eye with the same objective pattern of light that would emanate from the scene; if presented correctly the viewer would not be able to tell the difference between the picture and the reality it represents. But the technical problems of achieving this on a flat picture surface in a way that accommodates the full binocular visual field are considerable. We have shown that, under certain conditions, the artistic method discussed here is able to produce a more accurate representation of a given visual space than a set of geometrical perspective projections. This undermines the claim that geometrical perspective is the only accurate way to represent the visual world.

Our investigation of methods of representing the visual field is a preliminary and prompts several further lines of inquiry. For example, theorists of perspective have often argued that the perceived accuracy of geometrical projections depends on the viewing distance between viewer and picture (Malton, 1775; Leonardo da Vinci (in Kingslake, 1992; Kubovy, 1986; MacCurdy, 1954; Pirenne, 1970). On the basis of experiments conducted with our apparatus, but not reported here, we would expect to find that varying the viewing distance between participant and stimuli would significantly affect the accuracy ratings given to the different projections. It would also be interesting to modify the current experiments so that participants are prevented from looking anywhere other than the central disc in the dome and stimuli, perhaps by using eye tracker-triggered switches to blank the view if fixation strayed. It is possible participants glanced around the space, and this may have influenced their recall of its layout. And, finally, we do not yet know whether drawings of the appearance of the visual field made by other people with sufficient training would yield the same layout as that used in this study. Some provisional tests we have conducted suggest they would, but this is yet to be formally investigated.

At the current state of knowledge we are reliant on the skill and judgment of the artist when using our method to depict the phenomenal structure of visual space. It may be, however, that the psychological processes underpinning this phenomenology are amenable to geometrical modelling, and perhaps to being replicated in a three-dimensional rendering engine. This is a promising line of future research that could have implications for those interested in the structure of visual space. It may also prove useful for those wishing to produce computer-generated representations of the binocular visual field for applications in entertainment, communication, or simulation.

## 6 Conclusion

Our study showed that, under these conditions, the artistic depiction matched the appearance of the visual field more accurately than the geometrical perspective projections. We suggest this may be because the artistic depiction is closer to a representation of the apparent or subjective properties of the visual field than the objective properties of light or space. Given the limitations of geometrical projections when depicting wide angles of view on flat surfaces we suggest the artistic depiction is better adapted to these limitations, and so appears more naturalistic. Our results undermine the long-standing, widely advanced claim that geometrical perspective is the only accurate way to depict the visual world.
